# Evaluation of the effectiveness of a pressure injury prevention care bundle in an intensive care unit

**DOI:** 10.1186/s12912-026-04658-0

**Published:** 2026-04-22

**Authors:** Zehra Başayar, Gülay Yazıcı

**Affiliations:** 1 Vocational School of Health Services, Anesthesia Program, Ankara Medipol University, Ankara, Türkiye; 2 Faculty of Health Sciences, Department of Nursing, Ankara Yıldırım Beyazıt University, Ankara, Türkiye

**Keywords:** Care bundle, Pressure injury, Nursing, Intensive care unit

## Abstract

This study was conducted to evaluate the effectiveness of a pressure injury prevention care bundle in an intensive care unit. The study was designed as a quasi-experimental pretest–posttest study without a control group and was conducted between 01.10.2023 and 31.03.2024 in the Anesthesiology and Reanimation Intensive Care Unit of Bolu İzzet Baysal State Hospital. The study sample consisted of 28 nurses working in the intensive care unit and 108 hospitalized patients. The effect of the training provided to intensive care unit nurses on their compliance with the care bundle and on pressure injury (PI) incidence was evaluated. Data were collected using the “Nurses’ Identification Characteristics Form”, “Patients’ Identification Characteristics Form”, “Nurses’ Pressure Injury Knowledge Level Determination Questionnaire”, “Pressure Injury Prevention Care Bundle Control Form”, and the “Nurses’ Opinion Form on Pressure Injury Prevention Care Bundle”. In the evaluation of the data, descriptive statistics (number, percentage, mean ± standard deviation, and median [minimum–maximum]) were used. Comparisons between periods and PI rates were analyzed using the chi-square test, and *p* < 0.05 was considered statistically significant. When nurses’ compliance with the care bundle was examined, the mean care bundle compliance rate was 93.48 ± 10.26 in the October–December period and 95.33 ± 14.15 in the January–March period. Before the implementation of the study, the PI rate was 9.15 in the April–June 2023 period; however, this rate decreased to 2.72 in the January–March 2024 period, and the decrease in PI rate was statistically significant. As a result, it was determined that the training provided to nurses and the implementation of the evidence-based pressure injury prevention care bundle increased compliance with preventive interventions and reduced pressure injury rates. It is recommended that care provided for the prevention of pressure injuries in intensive care units should be planned under the guidance of a pressure injury prevention care bundle.

## Introduction

Pressure injury (PI), one of the priority areas of patient safety in healthcare services, is a preventable global health problem that increases the burden on healthcare systems. It is defined as “an injury localized to the skin and/or underlying soft tissue, usually over a bony prominence or associated with medical or other devices” [10, 20, 21]. Pressure injuries increase the length of hospitalization, healthcare costs, morbidity and mortality, and negatively affect the quality of life of patients and their families. It has been reported that the cost of treating a single full-thickness pressure injury can be extremely high. This indicates the significant economic burden associated with these injuries.

The EPUAP/NPIAP/PPPIA (2019) guidelines state that the prevalence of pressure injuries may vary widely between different geographical and clinical settings, ranging from 0% to 72.5% in healthcare settings. Worldwide, the average prevalence in healthcare settings is reported to be approximately 6%. A study conducted with 5,947 patients in palliative care units across several European countries reported a prevalence of pressure injuries of 18.1%. The study included data from Belgium, Portugal, Italy, Sweden, and the United Kingdom. When the results of different worldwide studies on the incidence of pressure injuries were compiled, it was reported that the global incidence ranged between 6% and 33.7%. Recent systematic reviews have emphasized that pressure injuries remain a significant patient safety problem in intensive care units and require evidence-based prevention strategies [18]. According to the results of a study conducted in Türkiye, data analyzed in 12 hospitals in different regions revealed an average pressure injury prevalence of 9.5%. In addition, according to the findings of the study, it was reported that 65.1% of the pressure injury cases developed in the hospital setting. In Türkiye, the prevalence of pressure injuries was found to be 12.7% for all clinical areas in the study conducted by Kaşıkçı et al. with 832 patients and 6% for internal and surgical clinics in the study conducted by İnan and Öztunç including 404 patients [3, 15, 16, 29].

Pressure injuries can occur in any area of healthcare delivery. According to studies conducted in various countries, the prevalence of pressure injuries varies between 7.8% and 13.5% in all clinical areas, 3%–18.5% in acute care clinics and 14%–33.7% in intensive care units (ICUs). Studies indicate that pressure injury rates are particularly high in ICUs. In a study compiling data from ninety countries and 1117 ICUs, it was reported that the prevalence of pressure injuries among ICU patients was 26.6%, while higher rates were observed among critically ill patients [17]. In a point prevalence study conducted in France, it was found that the prevalence of pressure injuries among ICU patients was 19%, while the prevalence of hospital-acquired pressure injuries was 12.5%. In studies conducted in Türkiye, it has been emphasized that the prevalence of pressure injuries in ICUs ranges between 15% and 65% [7, 9]. Patients followed up in ICUs are more prone to the development of pressure injuries due to factors such as limited activity and mobility, advanced age, vasopressor and sedation infusions, and changes in perfusion and nutritional status [4, 27].

Care bundles have been defined as a structured approach developed to improve patient care outcomes and care processes. A care bundle is also described as a set of evidence-based practices that improve the quality of healthcare services and patient safety when implemented together and consistently. Care bundles have been used in many clinical areas as well as in the prevention of pressure injuries ([2, 13, 24, 29]).

Although previous studies have examined pressure injury prevention strategies, studies evaluating the effectiveness of structured pressure injury prevention care bundles in intensive care units together with nurses’ knowledge levels and compliance with preventive interventions are limited. Therefore, evaluating the effectiveness of such interventions is important for improving patient safety and strengthening evidence-based nursing practice.

In this study, it was aimed to evaluate the implementation of the care bundle including the prevention interventions included in the guidelines four times a day by intensive care nurses who provide continuous patient care. As healthcare professionals responsible for implementing and sustaining care bundle interventions, intensive care nurses play a key role in preventing pressure injuries. They are expected to be aware that pressure injuries are preventable, to maintain up-to-date knowledge through regular training, and to effectively apply this knowledge in clinical practice.

### Hypotheses of the study

In order to achieve the aim of the study, the following hypotheses were proposed:

H0-1: Training on pressure injury prevention has no effect on nurses’ knowledge levels.

H0-2: Implementation of the “Pressure Injury Prevention Care Bundle” has no effect on reducing the pressure injury rate.

## Materials and methods

### Type of research

The study was conducted as a quasi-experimental pretest–posttest study without a control group in order to evaluate the effectiveness of the care bundle prepared for the prevention of pressure injuries in the intensive care unit.

### Place and time of the research

The study was conducted between 01.10.2023 and 31.03.2024 with nurses and inpatients in the Anesthesiology and Reanimation Intensive Care Unit of a state hospital in Türkiye.

### Population and sample of the study

The population of the study consisted of 30 nurses working in the Anesthesiology and Reanimation Intensive Care Unit of the relevant hospital between 01.10.2023 and 31.03.2024 and 216 patients hospitalized in the same unit.

To evaluate the nursing care provided in line with the care bundle, the following inclusion criteria were determined for patients:

- Patients hospitalized in the ICU
- Patients hospitalized for at least 24 h, as pressure injuries frequently occur within the first 24–48 h following immobilization
- Patients aged 18 years and older

Patients with contraindications such as cardiac arrest or brain death were excluded from the study. Patient follow-up was terminated in cases of discharge, transfer to another clinic or hospital, or death. The sample of the study consisted of 28 nurses and 108 patients who met the inclusion criteria and agreed to participate in the study.

### Development of a pressure injury prevention care package

The pressure injury prevention care bundle was developed by the researchers in accordance with the EPUAP/NPIAP/PPPIA (2019) guidelines and consisted of six parameters:

- Risk Assessment
- Skin Assessment
- Incontinence/Skin Care
- Activity Management
- Support Surface Use
- Nutritional Assessment

The care bundle form also included patient information such as name–surname, file number, age, gender, and diagnosis.

The form was evaluated by seven faculty members specialized in Surgical Nursing in terms of comprehensibility, formality, and scientific content. A scoring form including all items of the form was sent to the experts. The experts were asked to evaluate each item using a four-point scale based on the Content Validity Index (CVI) developed by Waltz and Bausell.

The content validity index for each item was calculated by dividing the number of experts who rated the item as 3 or 4 by the total number of experts. Based on the CVI analysis, the comprehensibility levels of the items were determined. According to the content validity ratios established by Veneziano and Hooper, the minimum acceptable value for seven experts at α = 0.05 significance level was 0.99.

### Training for pressure injury prevention

Training materials titled “Training Content for the Use of the Care Bundle in the Prevention of Pressure Injuries” and “Training Presentation for the Use of the Care Bundle in the Prevention of Pressure Injuries” were prepared by the researchers after reviewing the relevant literature [11, 21].

The training program included the following topics:

- Definition of pressure injury
- Epidemiology and risk factors
- Stages of pressure injury
- Pressure injury prevention interventions
- Care bundle concept
- Use of the care bundle in pressure injury prevention

The training content was evaluated by seven faculty members using a scoring form in order to assess the comprehensibility of the content and language.

### Data collection tools

#### Nurses’ descriptive characteristics form

This form was developed by the researchers based on the literature and consisted of 10 questions including gender, age, educational status, and duration of working in the intensive care unit [11, 21].

#### Patients’ descriptive characteristics form

This form consisted of 15 questions including gender, age, medical diagnosis, length of ICU stay, risk assessment scale score, and the presence of bowel movement during hospitalization [11, 21].

#### Questionnaire for determining nurses’ knowledge level on pressure injury

The questionnaire consisted of 20 multiple-choice questions covering four topics related to pressure injury prevention. Each correct answer was scored 5 points, and the maximum possible score was 100 [11, 21]. The content validity of the form was evaluated by seven experts in surgical nursing using the Content Validity Index (CVI) [26]. All items received ratings of 3 or 4, resulting in a CVI of 1.00, which exceeds the minimum acceptable value for seven experts [25]. Based on expert feedback, minor revisions were made and the final version of the form was established.

#### Pressure injury prevention care package control form

This form included patient information such as name–surname, file number, age, gender, and diagnosis. The form was completed by two nurses with intensive care experience who worked outside the ICU where the study was conducted, in order to ensure objective evaluation [11, 21].

#### Nurse opinion form for pressure injury prevention care package

Nurses’ opinions regarding the applicability of the care bundle were evaluated using a form consisting of 11 statements with response options of agree, undecided, and disagree [11, 21].

#### Preliminary implementation of the study

Before the main implementation, all nurses working in the ICU were trained by the first researcher on the use of the care bundle for pressure injury prevention. After the training, a 15-day pilot implementation was conducted in order for nurses to become familiar with the care bundle.

The pilot study was conducted with 10 patients hospitalized in the ICU, and at the end of the adaptation period, it was determined that nurses achieved 92.84% compliance with the care bundle.

#### Implementation of research

Ethical approval and institutional permissions were obtained before the implementation of the study. The responsible physician and nurse of the ICU were informed about the study process.

Between 01.08.2023 and 15.09.2023, the first researcher provided six training sessions to ICU nurses in the nurses’ room. Five training sessions were conducted with groups of five nurses and one session with three nurses.

Before the training, nurses were informed about the study and their written and verbal consent was obtained. The nurses were asked to code the “Questionnaire for Determining Nurses’ Knowledge Level on Pressure Injury” with a number to ensure anonymity.

At the end of the training, nurses completed the same questionnaire again using the same code. Each training session lasted approximately 60 min.

The nurses were instructed to apply the care bundle four times a day to patients meeting the inclusion criteria. ICU nurses evaluated patients according to the care bundle parameters and completed the Pressure Injury Prevention Care Bundle Control Form accordingly.

#### Evaluation of data

Descriptive statistics (frequency, percentage, mean, standard deviation, median, and minimum–maximum values) were used to summarize the data. The normality of continuous variables was assessed using the Shapiro–Wilk test, along with skewness and kurtosis values. Since the data did not meet the normality assumption, the Friedman test was used for repeated measurements, and the Mann–Whitney U test was used for comparisons between two independent groups. Statistical analyses were performed using SPSS version 25, and *p* < 0.05 was considered statistically significant.

A post-hoc power analysis was conducted using G*Power. Based on the Friedman test (χ² = 52.966), the effect size was calculated as W = 0.63 (*r* = 0.79, d = 1.30), indicating a large effect. Additionally, for the chi-square analysis (χ² = 67.240), the effect size was estimated as w ≈ 0.34, also indicating a large effect. These findings suggest that the statistical power of the study was high (> 0.90).

## Results

The nurses in the study were between 22 and 47 years of age and the mean age was 31.75 ± 7.22 years. Of the nurses, 64.29% (*n* = 18) were female, 46.43% (*n* = 3) were undergraduate graduates, 35.71% (*n* = 10) had 6–10 years of professional experience and 35.71% had 1–5 years of service in ICU. All of the nurses who participated in the study received training on IM, and 64.29% (*n* = 8) stated that they received this training during in-service training. It was found that 71.43% (*n* = 20) of the nurses needed training on the prevention and evaluation of IW, 50% (*n* = 10) of the nurses needed training on risk assessment, 20% (*n* = 4) on skin assessment, and 30% (*n* = 14) on pressure-distributing-reducing support surfaces. The nurses’ knowledge scores are presented in Table 1.

**Table 1:** Distribution of nurses’ knowledge scores after training

Knowledge Test Subject		Pre-test	Post-test	3 Months Later	6 Months Later	$\:{\boldsymbol{\chi\:}}^{2}$	*p*
Etiology and Risk Factors	Median	10.00	15.00	10.00	10.00	37.051^*^	**0.000**
	(Min-Max)	(0–20)	(0–25)	(0–20)	(0–25)		
	Rank Mean	2.07	3.25	1.96	2.71		
	$\:\stackrel{-}{X}$±ss	10.54 ± 4.38	14.29 ± 5.22	10.18 ± 5.00	12.50 ± 5.36		
Initiatives to Prevent PI	Median	15.00	15.00	15.00	15.00	26.734^*^	**0.000**
	(Min-Max)	(5–25)	(10–25)	(5–25)	(5–25)		
	Rank Mean	2.43	3.09	1.91	2,57		
	$\:\stackrel{-}{X}$±ss	15.18 ± 3.96	**16.96 ± 4.16**	13.75 ± 5.38	15.54 ± 4.38		
Staging of PI	Median	10.00	15.00	10.00	10.00	29.364^*^	**0.000**
	(Min-Max)	(0–25)	(5–25)	(0–25)	(0–25)		
	Rank Mean	2.34	3.05	2.27	2.34		
	$\:\stackrel{-}{X}$±ss	13.75 ± 6.03	16.07 ± 5.50	13.57 ± 6.06	13.75 ± 6.03		
Interventions for the Treatment and Care of PI	Median	0.00	5.00	5.00	5.00	8.42^*^	**0.000**
	(Min-Max)	(0–15)	(0–15)	(0–15)	(0–15)		
	Rank Mean	2.25	2.80	2.45	2.50		
	$\:\stackrel{-}{X}$±ss	**4.46 ± 5.83**	6.43 ± 5.42	5.00 ± 5.77	5.18 ± 5.69		
General Knowledge Score	Median	45.00	55.00	45.00	50.00	52.966	**0.000**
	(Min-Max)	(20–65)	(30–80)	(20–70)	(20–80)		
	Rank Mean	1.91	3.75	1.66	2.68		
	$\:\stackrel{-}{X}$±ss	43.93 ± 10.83	**53.75 ± 11.35**	42.50 ± 11.90	46.96 ± 12.50		

$\:{\chi\:}^{2}$: Chi-square value (Firedman Test); ^*^p < 0.05

It was seen that the nurses’ pre-test, post-test, three-month post-test, three-month post-test and six-month post-test measurements of the knowledge test on the etiology and risk factors, interventions for prevention of PI, staging of PI, interventions for treatment and care of PI showed a statistically significant difference (*p* < 0.05). It was determined that the total scores of the nurses changed according to time (χ2 = 52.966, *p* < 0.001). When the knowledge test achievement scores of the nurses were examined, it was seen that the pre-training knowledge scores were significantly lower than the post-training scores (*p* < 0.05). Post-training 3rd month knowledge scores were significantly lower than pre-test, post-test and 6th month knowledge scores (*p* < 0.05).

**Table 2:** Distribution of nurses’ opinions on pressure injury prevention care package

WIEWS	Agree	Undecided	Disagree
	*n* (%)	*n* (%)	*n* (%)
Evidence-based care is provided.	**26 (92.8)**	2 (7.2)	-
Parameters are adequate for the prevention of PI.	**26 (92.8)**	2 (7.2)	-
Positively affects clinical decision-making skills.	**24 (85.7)**	2 (7.1)	2 (7.1)
There is no obstacle in its implementation.	**20 (71.4)**	5 (17.8)	3 (10.7)
Easy to use.	**18 (64.2)**	2 (7.1)	8 (28.5)
Its use in intensive care is necessary.	**19 (67.8)**	5 (17.8)	4 (14.2)
I experienced obstacles due to lack of personnel.	5 (17.8)	5 (17.8)	**18 (64.2)**
I experienced obstacles due to lack of time.	10 (35.7)	4 (14.2)	**14 (50)**
I experienced obstacles due to lack of materials.	5 (17.8)	5 (17.8)	**18 (64.2)**
I experienced obstacles due to the unstable condition of the patients.	10 (35.7)	4 (14.2)	**14 (50)**
I experienced obstacles due to patients’ refusal of the application.	-	2 (7.2)	**26 (92.8)**

92.8% (*n* = 26) of the nurses stated that it would be possible to provide an evidence-based, safe health care service to patients with the implementation of the PIPCP and that the parameters included in the PIPCP were sufficient for the prevention of HAI. On the other hand, 85.7% of the nurses (*n* = 24) stated that the use of the PIPCP would positively affect the clinical decision-making skills of nurses, as shown in Table 2.The patients were between 18 and 102 years of age and the mean age was 69.69 (± 18.41) years. 47.22% (*n* = 51) of the patients were female, mean PI was 28.69 (± 6.71) and 45.37% (*n* = 49) were obese. 75.93% (*n* = 82) of the patients had chronic diseases. The most common chronic diseases were COPD 30.86% (*n* = 25), DM + HT 22.22% (*n* = 18) and DM 20.99% (*n* = 17). The frequency of fecal incontinence was once for 81.48% (*n* = 88) of the patients. Urinary continence was not observed in 83.33% (*n* = 90) of the patients. 58.33% (*n* = 63) of the patients had PI at the time of hospitalization. Mean hemoglobin level was 9.16 (± 1.79), mean albumin level was 22.07 (± 3.81) and mean blood glucose level was 155.21 (± 54.09).

**Table 3:** Distribution of pressure injury development according to characteristics

	Characteristics	n	%
PI Phase	Phase I	8	**61.53**
	Phase II	3	23.07
	Suspected Deep Tissue Damage	2	15.38
PI Area	Sacrum+Gluteal+Coxix	8	**61.53**
	Heel	2	15.38
	Scapula	1	7.69
	Tibia	1	7.69
	Femoral Head	1	7.69
Width*Length*Depth	1*1*1	8	**61.53**
	2*2*1	3	23.07
	2*3*1	1	7.69
	Total	13	100.0

When the development status of the patients within the scope of the study was analyzed, it was seen that 12.03% of the patients had developed BC. It was found that 61.53% (*n* = 8) of the patients developed stage 1, 23.07% (*n* = 3) developed stage 2 and 15.38% (*n* = 2) developed suspected deep tissue injury. According to the distribution of the patients according to the site of the PI, 61.53% (*n* = 8) developed in the sacrum + glutel + coccyx, 7.69% (*n* = 1) in the scapula, 7.69% (*n* = 1) in the tibia, and 7.69% (*n* = 1) in the femoral head. When the distribution according to width-height-depth was analyzed, it was found that 61.53% (*n* = 8) were 1*1*1, 23.07% (*n* = 3) were 2*2*1 and 7.69% (*n* = 1) were 2*3*1. The distribution of pressure injury characteristics is presented in Table 3.

**Table 4:** Distribution of the nurses’ compliance over time

		October-November-December		January-February-March		Total		Test
		n	%	n	%	n	%	P
General Package Compatibility	Incompatible	55	6.52	38	4.67	93	5.62	$\:{\chi\:}^{2}$=2.673 *p* = 0.102
	Compatible	788	93.48	775	95.33	1563	94.38	
	Total	843	100	813	100	1656	100	
Risk Assessment	Incompatible	30	3.55	18	2.21	48	2.89	$\:{\chi\:}^{2}$=2.673 *p* = 0.102
	Compatible	814	96.45	797	97.79	1611	97.11	
	Total	844	100	815	100	1659	100	
Skin Evaluation	Incompatible	26	3.10	16	1.98	42	2.55	$\:{\chi\:}^{2}$=2.096 *p* = 0.148
	Compatible	812	96.90	793	98.02	1605	97,45	
	Total	838	100	809	100	1647	100	
Incontinence/Skin Care	Incompatible	20	2.39	15	1.85	35	2.13	$\:{\chi\:}^{2}$=0.561 *p* = 0.454
	Compatible	818	97.61	794	98.15	1612	97.87	
	Total	838	100	809	100	1647	100	
Activity Management	Incompatible	34	4.06	15	1.85	49	2.98	$\:{\chi\:}^{2}$=6.921 ***p* = 0.009**
	Compatible	804	95.94	794	98.15	1598	97.02	
	Total	838	100	809	100	1647	100	
Support Surface Usage	Incompatible	39	4.65	28	3.46	67	4.07	$\:{\chi\:}^{2}$=1.501 *p* = 0.221
	Compatible	799	95.35	781	96.54	1580	95.93	
	Total	838	100	809	100	1647	100	
Nutrition Assessment	Incompatible	14	1.67	14	1.74	28	1.71	$\:{\chi\:}^{2}$=0.010 *p* = 0.920
	Compatible	822	98.33	792	98.26	1614	98.29	
	Total	836	100	806	100	1642	100	

A statistically significant difference was determined when the compliance measurements of the nurses’ compliance with the general activity management theme of the PI prevention care package were evaluated according to time (*p* < 0.05). For the activity theme, the compliance rate in the first period (October-November-December 2023) was 95.94% (*n* = 108), while the compliance rate in the second period (January-February-March 2024) was 98.15% (*n* = 794). The compliance rate in the second period is higher than the compliance rate in the first period as shown in Table 4.

**Table 5:** Distribution of pressure injury rates before and after pressure injury care package

	Before Study		Period of Study			
	April-May-June/2023	July-August-September/2023	October-November-December/2023	January-February-March/2024		
	**n (Rate)**	**n (Rate)**	**n (Rate)**	**n (Rate)**	$\:{\boldsymbol{\chi\:}}^{2}$	**P**
PIPCP	13 (9.15)	14 (7.85)	9 (5.98)	4 (2.72)	67.240	0.000

For the six months before and the six months after the implementation of the pressure injury prevention care package, the rates were 9.15, 7.85, 5.98 and 2.72 for the periods April-May-June 2023, July-August-September 2023, October-November-December 2023 and January-February-March 2024, respectively, as shown in Table 5. As a result of the chi-square analysis, it was determined that there was a decrease in the PI rate in the January-March 2024 period compared to the July-September 2023 and October-December 2023 periods; there was a decrease in the October-December 2023 period compared to the April-June 2023 and July-September 2023 periods, but the decrease was not statistically significant. The decrease in the PI rate in the period January-March 2024 compared to the period April-June 2023 was statistically significant (*P* < 0.05).

## Discussion

Prevention of pressure injuries is considered an important indicator of the quality and effectiveness of nursing care. Practices required to prevent pressure injury development include holistic risk assessment, regular skin assessment, nutritional support, activity management, moisture/incontinence management, and appropriate support surface use. Implementing these interventions through structured care bundles and providing periodic training to nurses responsible for patient care may contribute to improving the quality of nursing care and patient safety [5, 8, 12, 17, 19, 22, 28].

In the present study, nurses’ knowledge scores regarding pressure injury prevention changed over time. The mean knowledge score increased significantly from 43.93 before training to 53.75 after training (*p* < 0.05). However, the mean score decreased to 42.50 at the third month after training and increased again to 46.96 at the sixth month. These findings indicate that training programs may improve nurses’ knowledge levels in the short term, but periodic reinforcement may be necessary to maintain knowledge over time. Therefore, the H0-1 hypothesis was rejected.

Similar findings have been reported in previous studies. Sezgünsay et al. [23] reported a significant relationship between nurses’ participation in educational activities such as training programs, courses, and symposiums related to pressure injury prevention and their level of knowledge. In other studies, nurses who participated in in-service training programs on pressure injury prevention demonstrated higher knowledge scores compared with those who did not participate in such programs. Consistent with these findings, the results of the present study highlight the importance of providing regular educational interventions to improve nurses’ knowledge, skills, and attitudes regarding pressure injury prevention [6, 18, 23].

In this study, nurses’ compliance with the pressure injury prevention care bundle was evaluated in two different periods. The mean compliance rate was 93.48 ± 10.26 in the October–December 2023 period and 95.33 ± 14.15 in the January–March 2024 period. Based on a total of 1656 patient evaluations, the overall mean compliance rate of nurses during the six-month period was 94.38 ± 12.92. High compliance rates may increase the effectiveness of care bundle interventions and improve the likelihood of achieving the desired outcomes in patient care [14].

Previous studies have also demonstrated that care bundle implementation may contribute to reducing pressure injury incidence. In the study conducted by Arıcan [1], the average care bundle compliance increased from 91.39 in the April–June 2018 period to 93.77 in the July–September 2018 period. In that study, pressure injury development was observed in 12 of 70 patients in the intervention group compared with 37 of 70 patients in the control group. Similarly, Tayyib et al. [24] reported that pressure injury incidence decreased significantly from 32.86% to 7.14% following care bundle implementation.

Consistent with the findings reported in the literature, the results of the present study also showed a decrease in pressure injury rates during the study period. In the first stage of the study, the pressure injury rate was 7.85%. During the data collection stage, the pressure injury rate was 6% with a care bundle compliance rate of 93.48%. In the final stage of the study, the pressure injury rate decreased to 2.72% while the care bundle compliance rate increased to 95.33%. When the overall results were evaluated, it was observed that as compliance with the care bundle increased, pressure injury rates decreased. In addition, when the pressure injury rates during the study period were compared with those recorded during the six months before the study, the pressure injury rate decreased from 9.15 in the April–June 2023 period to 2.72 in the January–March 2024 period, and this decrease was statistically significant (*p* < 0.05). Therefore, the H0-2 hypothesis was rejected.

When the sub-parameters included in the care bundle were examined (risk assessment, skin assessment, incontinence/skin care, activity management, nutritional assessment, and support surface use), the highest level of non-compliance was observed in the incontinence/skin care parameter. In the October–December period, barrier products were not used in 5.25% (*n* = 44) of patients at risk of moisture-related skin damage. In the January–March period, this rate decreased to 3.58% (*n* = 29). Similar findings were reported in the study conducted by Arıcan [1], where skin assessment was not performed in 7.4% of cases and barrier products were not used in 4.4% of patients to protect the skin from moisture-related damage.

These findings should be interpreted cautiously due to the quasi-experimental design of the study.

## Conclusion

This study evaluated the effectiveness of implementing a pressure injury prevention care bundle and its effect on nurses’ knowledge levels regarding pressure injury prevention. The results showed that nurses’ knowledge levels increased after training, although a decrease was observed in the third month, suggesting the need for continuous educational reinforcement.

In addition, nurses’ compliance with the pressure injury prevention care bundle was found to be high during the study period. The high compliance rates were accompanied by a decrease in pressure injury rates observed during the implementation period. These findings suggest that implementing structured care bundle interventions supported by nurse training may contribute to improving pressure injury prevention practices in intensive care units.

In this context, it is recommended that regular training programs including up-to-date information be implemented to enhance nurses’ knowledge of pressure injuries, and that the use of care bundles for pressure injury prevention—facilitating the integration of evidence-based practices into clinical settings—be expanded in intensive care units.

### Limitations

No sampling procedure was performed in this study. The research was conducted including all nurses working in the intensive care unit during the six-month study period and all patients who met the inclusion criteria. In our country, pressure injury rates are generally monitored using three-month and six-month periods. Accordingly, the pressure injury rates obtained during the six-month study period were compared with those of the previous period.

## Data Availability

The datasets generated and/or analysed during the current study are not publicly available due to privacy and ethical restrictions but are available from the corresponding author on reasonable request.
